# Pattern of cancers among adolescents and young adults seen at a radiation oncology clinic in Nigeria

**DOI:** 10.3332/ecancer.2025.2019

**Published:** 2025-10-17

**Authors:** Abbas A Abdus-salam, Chiamaka G Ehiedu, Olabisi T Ojo, Atara I Ntekim, Mutiu A Jimoh, Mariam A Bashir, Daluchukwu J Obi, Abel S Anegbe

**Affiliations:** 1 Department of Radiation Oncology, University of Ibadan, Ibadan 200005, Nigeria; 2 Department of Radiation Oncology, University College Hospital, Ibadan 200212, Nigeria; 3 Department of Radiation Oncology, Lagos University Teaching Hospital, Lagos 100254, Nigeria

**Keywords:** adolescents, young adults, cancer

## Abstract

**Background:**

Cancers in adolescents and young adults (AYAs; ages 15–39 years) differ from those in children and older adults. AYAs with cancers have received less research focus over the years despite their unique medical, psychological, socioeconomic and sexual concerns. The burden of cancer among this age group in Nigeria is unknown. This study aims to determine the pattern of cancers in AYAs seen in a radiation oncology clinic in Nigeria.

**Method:**

A retrospective study among AYAs (15–39 years) with cancer who presented at a Radiation Oncology Clinic in Nigeria between January 2018 and December 2023.

**Results:**

There were 529 new cancer cases among AYAs, accounting for 14.1% of all cancer cases seen during the period. The male-to-female ratio was 1:3. The majority (72.8%) were in the 30–39 age range, with a mean age of 32.06 ± 6.18 years. Females had a higher mean age (33.01 ± 5.31 years) than males (29.43 ± 7.57 years) (*p* < 0.001). Breast (39.1%), bone and soft tissue (12.9%) and cervix (11.6%) were the most common sites of cancer in AYAs. Among the males, bone and soft tissue (20.0%), nasopharynx (13.6%) and sinonasal (10.7%) were the most common sites, while breast (52.2%), cervix (15.4%) and bone and soft tissue (10.0%) were the most common sites among females.

**Conclusion:**

AYAs accounted for 14.1% of all new cancer cases. Breast cancer was the most common cancer among female AYAs, while bone and soft tissue cancers were the most common among males.

## Introduction

Adolescent and young adult (AYA) cancer patients are individuals who, at the time of cancer diagnosis, are aged 15–39 years [1]. This group of cancer patients are unique due to their peculiar psychosocial and economic concerns (education, employment, romantic relationships, fertility, parenting and financial/insurance issues), cancer biology, risk factors (intrinsic and extrinsic), delayed diagnosis (due to insufficient awareness of the possibility of cancer in this age group), low rates of participation in clinical trials and survivorship concerns (medical, physical and psychosocial late side effects) [1–4].

Common cancers seen in AYAs include haematological malignancies, sarcomas, breast cancers, brain tumours, melanomas, colorectal cancers, thyroid cancer and testicular cancers [3, 5]. The spectrum of cancer types that occur in AYAs is unique [1] and varies based on the age of the patients [2, 3].

Over the years, there has been an increase in the incidence of cancers among AYAs [5, 6], with little improvement in survival rates, which are modest compared to improvements in survival rates in other age groups [3].

In Nigeria, there is a scarcity of data on AYA oncology patients, which is needed to develop policies and guidelines that would help improve cancer survival in this population. This study aims to describe the incidence and pattern of cancers in AYAs presenting at a radiation oncology clinic in a tertiary hospital in Nigeria.

## Methods

A descriptive retrospective study of AYAs (15–39 years) with histopathologically confirmed cancers, who presented at the Department of Radiation Oncology, University College Hospital, Ibadan, between January 2018 and December 2023, was done. Patients' age, sex, histologic diagnosis, cancer site and year of presentation were extracted from the Radiation Oncology Clinic's new patient register with a proforma. Data were collated and analysed using SPSS version 24. Results were presented in prose, tables, charts and graphs. The student’s *t*-test was used to compare quantitative variables, and the chi-squared test was used to test for association between categorical variables. Ethical approval was obtained from the joint ethical review committee of the University of Ibadan and University College Hospital, Ibadan (UI/EC/25/0262).

## Results

**Figure 1.**
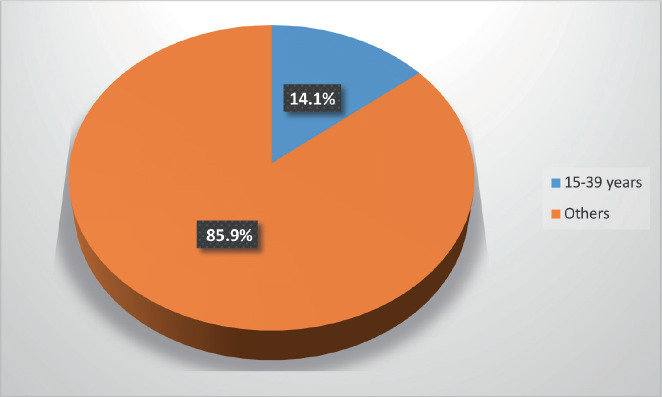
Age range at presentation.

A total of 3,761 new patients with cancer diagnoses presented at the Radiation Oncology Clinic, UCH, Ibadan, from January 2018 to December 2023. Among them were 529 patients (14.1%) who fall within the AYAs (15–39 years) category (Figure 1).

**Figure 2.**
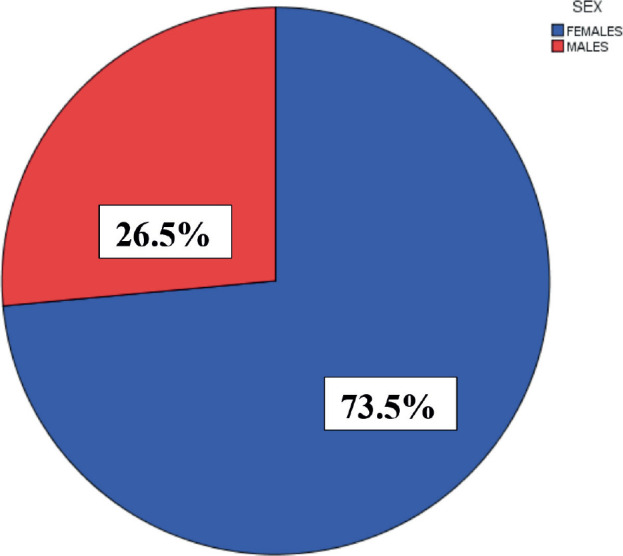
Sex distribution of AYA.

**Figure 3.**
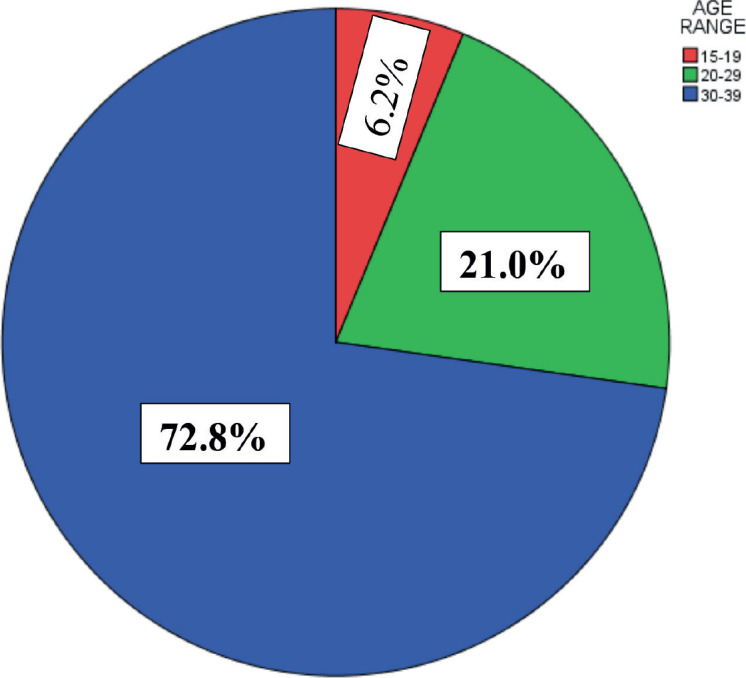
Age distribution of AYAs.

Among the AYAs, almost three-quarters (73.5%) were females, giving a male-to-female ratio (M:F) of 1:3. A large majority of them (72.8%) were within the age group 30–39 years (Figures 2 and 3), while their mean age was 32.06 ± 6.18 years. There was a statistically significant difference between the mean ages of males (29.43 ± 7.57 years) and females (33.01 ± 5.31 years) with *p* < 0.001 and there was a statistically significant association between age group and sex as the proportion of males within the age group 15–19 years (15.7%) was higher than that of the females (2.8%) and the proportion of females within the age group 30–39 years (78.1%) was higher than the proportion for males (57.9%) with *p* < 0.001 (Table 1).

**Figure 4.**
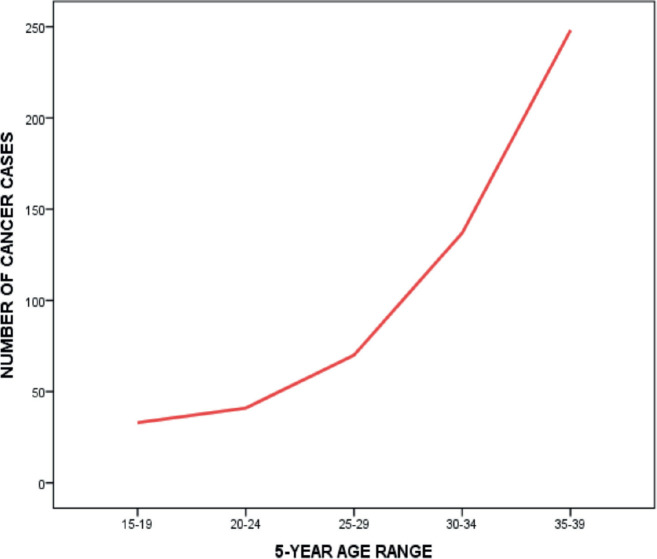
Cancer cases in AYAs by age group.

**Figure 5.**
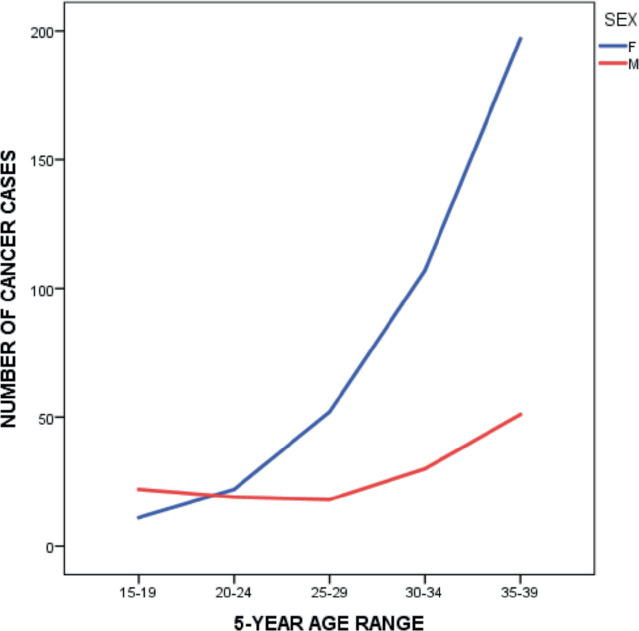
Cancer cases in AYAs by sex and age group.

There was a rapid rise in the number of cancer cases among AYAs from the 25–29 years age group, with the peak among those within 35–39 years (Figure 4). Among female AYAs, a sharp rise was noticed after the age group 20–24 years; however, among the males, a less steep rise occurred after the age group 25–29 years, following a decline after the age group 15–19 years (Figure 5).

**Figure 6.**
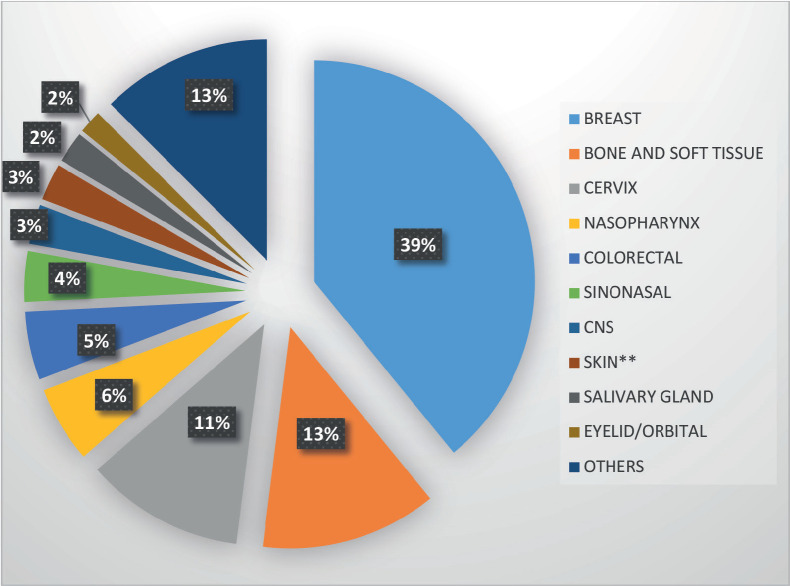
Leading sites of new cancer cases in AYAs.

**Figure 7.**
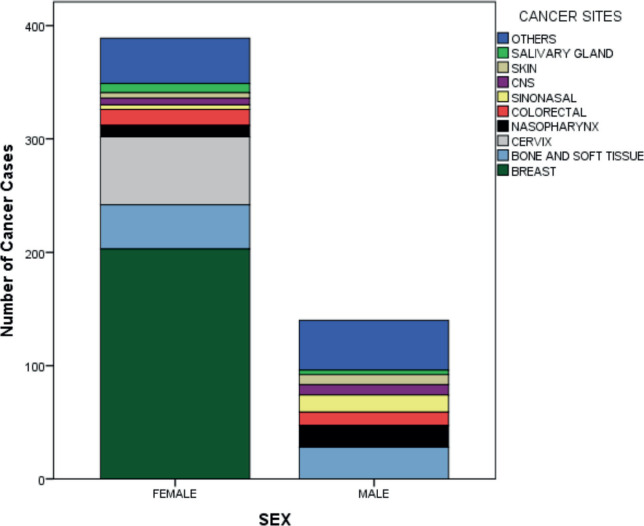
Cancer cases in AYAs by site and sex.

**Figure 8.**
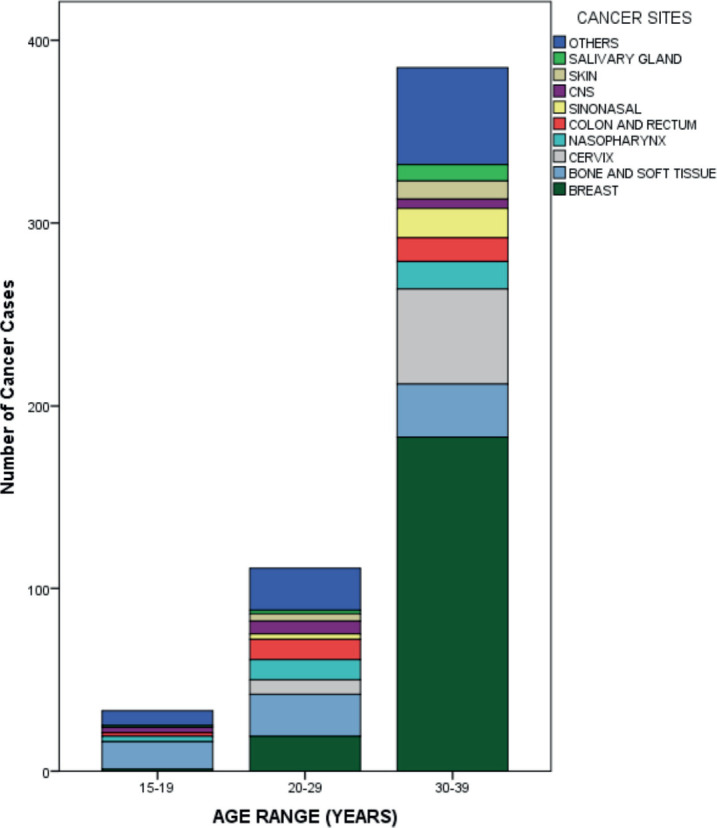
Case distribution in AYAs by age group.

**Figure 9.**
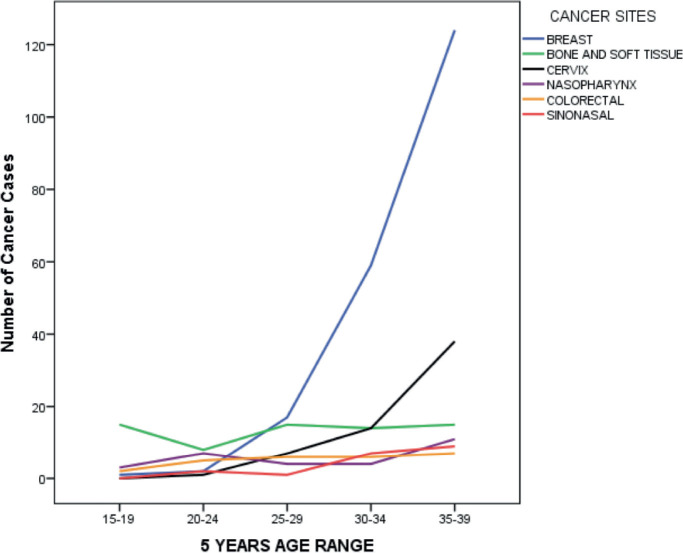
Cancer cases in AYAs by site and age group.

Breast accounted for almost two-fifths (39.1%) of AYAs' cancer sites, followed by bone and soft tissue (12.9%), cervix (11.6%), nasopharynx (5.6%) and colorectal (5.0%). (Table 2 and Figure 6) Among the male AYAs, bone and soft tissue accounted for a fifth (20.0%) of the cancer sites, followed by nasopharynx (13.6%), sinonasal (10.7%), colorectal (8.6%), central nervous system (CNS) (6.4%) and skin (6.4%). Among female AYAs, the breast was the most common site, accounting for 52.2% followed by cervix (15.4%), bone and soft tissue (10.0%), colorectal (3.6%) and nasopharynx (2.6%). (Table 3 and Figure 7) Among AYAs within 15–19 years, bone and soft tissue (46.9%), CNS (9.4%), nasopharynx (9.4%), colorectal (6.3%) and lip and oral cavity cancer (6.3%) were the most common cancer sites. Bone and soft tissue (21.5%), breast (17.8%), colorectal (10.3%), nasopharynx (10.3%) and cervix (7.5%) were the most common cancer sites among AYAs between 20 and 29 years, while breast (48.2%), cervix (13.7%), bone and soft tissue (7.6%), sinonasal (4.2%) and nasopharynx (3.9%) were the most common sites of cancers among AYAs between 30 and 39 years (Table 4 and Figure 8). The incidence of common cancers in AYAs increased with increasing age (Figure 9 and Table 5).

## Discussion

In Nigeria, 83,197,647 persons were within the AYA age group (15–39 years), accounting for 38.4% of the population in 2022, with a M:F ratio of ≈ 1:1 [7]. This group constitutes almost two-fifths of the Nigerian population, but studies about cancers within this age group are scarce in Nigeria, hence the need for this study.

This study revealed that there were 529 cancer cases among AYAs, accounting for 14.1% (≈ 1 in 7) of all new cancer cases seen within the period under review. This finding is in tandem with results from studies done by Darling *et al* [8] and Kakkar *et al* [9], who reported that 12% and 15% of new cancer cases in India occurred in AYAs, respectively. In contrast to our finding, Trama *et al* [6] reported that AYA cancers accounted for 5% of all new cancer cases in Europe in 2020, while the National Cancer Institute estimates revealed that AYA cancers accounted for 4.2% of new cancer cases in the United States of America (USA) in 2024 [5]. The difference between our findings and those from Europe and the USA could be because some common cancers, such as breast cancer, have been shown to occur at a younger age in blacks compared to whites [10]. Additionally, a comparison of the populations of Nigeria (2022) and the USA (2023) showed that the percentage of people in the 15–39 age range was marginally greater in Nigeria than in the USA (38.4% versus 33.4%) [7, 11]. The high prevalence of breast cancer and cervical cancer may also have skewed our figures in comparison to Western countries, where these cancers are better controlled.

This study demonstrated that 73.5% of AYAs with cancer were female, whereas 26.5% were male, indicating a female preponderance, with a M:F ratio of ≈ 1:3. This difference is due to the sex distribution of breast cancer, which is the most common cancer in Nigeria [12]. A study done in Europe also revealed a female preponderance with a M:F ratio of 1:2 [6]. Additionally, a global study of cancers in AYAs reported a M:F ratio of ≈1:2 [13]. However, studies from Indian reported a slight male preponderance [8, 9].

The mean age of AYAs in this study was 32.1 ± 6.2 years. Talukder *et al* [14] also demonstrated similar findings. They reported that 34.5 ± 6 years was the mean age of AYAs with cancer in India. In our study, AYA cancer occurred earlier in males compared to females, as depicted by the mean ages, which were significantly lower among male AYAs compared to females (29.4 ± 7.6 years versus 33.0 ± 5.3 years, *p* < 0.001). AYAs within the 30–39 age group accounted for the majority (72.8%) of patients in our study. This is supported by other studies, which reported that 65.2%–68.3% of AYA cancers occurred among those 30–39 years of age [4, 8, 9]. Worthy of note in our study is that the proportion of male AYAs with cancer within the age group 15–19 and 20–29 years was higher compared to the proportion of females within same age range (15.7% versus 2.8% and 26.4% versus 19.0%, respectively) while the proportion of females within the age group 30–39 years was higher compared to the males (78.1% versus 57.9%, *p* < 0.001). Also, there was a switch from male preponderance among AYAs with cancer within age 15–19 years (M:F = 2:1) to female preponderance among those 20–29 and 30–39 years (M:F = 1:2 and ≈1:4, respectively). These further buttress the early onset of AYAs in males compared to females, and this could be due to the age distribution of frequently occurring cancers as well as their risk factors in both sexes.

The incidence of AYA cancers increased with increasing age, with almost half (46.9%) occurring among those between 35 and 39 years (males: 36.4%, females: 50.6%). Miller *et al* [4] and Kakkar *et al* [9] also demonstrated similar findings in the USA and India. Compared to the females who had a sharp, steady increase in incidence with increasing age, there was an initial slight decline in the incidence among the males within age groups 20–24 and 25–29 years before a gradual increase.

This study showed that breast cancer was the most common malignancy in AYAs, followed by bone and soft tissue cancers, cervical cancer and nasopharyngeal cancer. In Europe and the USA, breast cancer, thyroid cancer, testicular cancer and skin melanoma were the most common cancers seen in AYAs [5, 6], while breast cancer, thyroid cancer, astrocytoma and gastric cancer were the most common cancers in AYAs in India [8]. The second to fourth most common cancers in our study differed from the reports from Europe, the USA and India, and this could be due to different genetic, sociodemographic, environmental, lifestyle and health care system factors, which should be evaluated. This is supported by findings reported by Li *et al* [13] who demonstrated that breast cancer and thyroid cancer were the most common AYA cancers in very high and high Human Development Index (HDI) countries, while breast cancer and cervical cancer were the most common AYA cancers in medium and low HDI countries. This is not unexpected, as medium to low HDI countries have been associated with a high burden of poverty and infection-related cancers [15]. Global cancer statistics for AYAs revealed that breast cancer, thyroid cancer and cervical cancer were the most commonly diagnosed cancers in AYAs [13]. This reflects a combined picture of the most common cancers in AYAs in high, medium and low HDI countries. The distribution of AYA cancers differed based on sex in our study. Bone and soft tissue cancers (20.0%), nasopharyngeal cancer (13.6%) and sinonasal cancer (10.7%) were the most common AYA cancers among males, while breast cancer (52.2%), cervical cancer (15.4%) and bone and soft tissue cancers (10.0%) were the most common among females. A Nigerian study by Ntekim *et al* [16] also demonstrated similar findings among female AYAs with cancer. They reported breast cancer (49.75%) and cervical cancer (7.33%) to be the most common malignancies among female AYAs [16]. AYA cancers also differed based on sex in a study done in USA which reported breast cancer (22.8%), thyroid cancer (21.7%) and melanoma (9.5%) as the most common cancers among female AYAs and testicular cancer (20.0%), lymphoma (15.6%) and melanoma (8.4%) as the most common cancers among male AYAs [17]. The incidence rates of thyroid cancer, lymphoma and melanoma were higher in the USA study compared to our study. This could be due to the lower incidence of these malignancies in the Nigerian population [12]. The burden of these malignancies could also be underestimated due to limited access to appropriate diagnostic facilities (such as thyroid ultrasound scan) and a trained technical workforce. It could also be because patients with thyroid cancer and lymphoma usually present to the surgical oncologist and haemato-oncologist, respectively, in our setting, hence their under-representation in our study. AYA cancers also varied across age groups in our study. Bone and soft tissue cancers, CNS tumours and nasopharyngeal cancers were the most common among AYAs between 15 and 19 years. Bone and soft tissue cancers, breast cancer and colorectal cancer were the most common among AYAs 20–29 years, while breast cancer, cervical cancer and bone and soft tissue cancers were the most common among AYAs 30–39 years. Miller *et al* [4] also revealed differences in AYA cancers across age groups. They reported thyroid cancer, Hodgkin’s lymphoma and CNS tumours as the most frequent cancers among AYAs within 15–19 years, thyroid cancer, testicular cancer and germ cell tumours as the most frequent cancers among AYA within 20–29 years and breast cancer, thyroid cancer and melanoma as the most frequent cancers among AYA within 30–39 years [4]. Li *et al* [13] demonstrated that leukaemia and thyroid cancer were the most prevalent cancers in adolescents, and breast and thyroid cancer were the most common among individuals in the 20–39 age range.

Breast cancer was the most common cancer among AYAs (39.1%), female AYAs (52.2%) and AYAs between 30 and 39 years (48.2%). AYAs accounted for 17.1% of all new breast cancer cases seen within the period under review. All breast cancers in AYAs occurred in females, with a mean age of 34.6 ± 3.8 years, an age range of 18–39 years and a peak age of 35 years. There was a sharp increase in the incidence of breast cancer from AYAs within the age group 20–24 years, and 90.1% of AYA breast cancer occurred among those within 30–39 years.

Bone and soft tissue cancers were the second most common cancer among AYAs (12.9%) and the most common cancer among male AYAs (20.0%), AYAs between 15 and 19 years (46.9%) and AYAs between 20 and 29 years (21.5%). AYA accounted for 32.1% of all sarcomas (bone and soft tissue) seen within the period of the study. The M:F ratio was 1:1.4, the mean age of occurrence was 27.5 ± 7.6 years, the age range was between 15 and 39 years and most occurred at 26 years. The incidence remained fairly stable across a 5-year age range after a slight decline among those within 20–24 years.

Cervical cancer was the third most common cancer among AYAs (11.6%) and the second most common malignancy among female AYAs (15.4%) and AYAs within 30–39 years (13.7%). AYA cervical cancer accounted for 6.7% of all new cases of cervical cancer. The mean age was 34.8 ± 3.8 years, the age range was between 23 and 39 years and the peak age was 39 years. The incidence steadily increased with an increasing 5-year age range till 30–34 years, after which a sharp increase occurred.

Nasopharyngeal cancer was the fourth most common cancer among AYAs (5.6%) and the second most common cancer among male AYAs (13.6%). 29% of all nasopharyngeal cancers occurred in AYAs. The M:F ratio was 2:1, the mean age of occurrence was 29.2 ± 7.3 years and the age range was 19–39 years. The incidence remained fairly stable across a 5-year age range till the 30–34 years age group, after which a slight increase occurred.

Colorectal cancer was the fifth most common AYA cancer (5.0%) and the third most common malignancy among AYAs between 20 and 29 years (10.3%). About a quarter (23.4%) of all new cases of colorectal cancer occurred in AYAs. The M:F ratio was 1:1.2, the mean age of occurrence was 29.2 ± 6.3 years and the age range was 18–38 years. The incidence increased slightly after the age group 15–19 years and remained stable thereafter.

Sinonasal cancer was the sixth most common AYA cancer (3.7%) and the third most common in male AYAs (10.7%). About a fourth (24.1%) of all new sinonasal cancers occurred in AYAs. The M:F ratio was 4:1, the mean age of occurrence was 32.7 ± 5.6 years, the age range was 20–39 years and the peak age was 37 years. The incidence increased gradually with increasing age.

## Study limitations

This study was retrospective and thus was limited by the occurrence of some missing data. The hospital-based nature of this study should also be considered before drawing any conclusions/generalisations. Some cancers not typically treated with radiotherapy may be underrepresented in this study, as the findings of this study were based on data from a single radiation oncology clinic. Furthermore, there is a high probability of referral bias in this study due to limited access to cancer diagnosis and treatment facilities, especially in rural communities. Survival analysis was also not done. A population-based longitudinal study with survival analysis would provide a better understanding of the epidemiology of cancer in AYAs. Despite these limitations, this study highlighted the incidence and pattern of cancers in AYAs.

## Conclusion

AYA cancers accounted for 14.1% (≈1 in 7) of cancer cases. Breast cancer and sarcomas (bone and soft tissue) were the most common malignancies among female and male AYAs, respectively. The distribution of AYA cancers varied across sexes and age groups. AYA is a peculiar group of individuals with unique cancer epidemiology and varying distribution across sexes and age groups. There is a need for focused attention on AYAs as well as AYAs with cancer to provide targeted intervention across all levels of cancer control for this population. The AYAs represent a particularly productive category of the national population, and addressing their health challenges has the propensity to improve national productivity.

## Conflicts of interest

The authors declare that they have no conflicts of interest.

## Funding

None.

## Author contributions

All authors contributed to the conception and design of the study. All authors took part in the management of the patients. Data collection was done by Chiamaka G Ehiedu and Olabisi T Ojo under the supervision of Abbas A Abdus-Salam. Data analysis was done by Chiamaka G Ehiedu and reviewed by all authors. The first draft of the manuscript was written by Chiamaka G Ehiedu and Olabisi T Ojo, and all authors revised and commented on several versions of the manuscript. The final manuscript represents the aggregate of revisions, adjustments and approval by all the authors.

## Figures and Tables

**Table 1.** Mean age and age range by sex.

	Sex		
	Males	Females	*p*-value
**Mean age**	29.43 ± 7.573	33.01 ± 5.307	<0.001*
**Age range**			
15–19	22 (15.7%)	11 (2.8%)	<0.001**
20–29	37 (26.4%)	74 (19.0%)	
30–39	81 (57.9%)	304 (78.1%)	

*T-test. **Chi-square

**Table 2.** Leading sites of new cancer cases in AYAs.

AYAs*	
Breast	203 (39.1%)
Bone and soft tissue	67 (12.9%)
Cervical	60 (11.6%)
Nasopharynx	29 (5.6%)
Colorectal	26 (5.0%)
Sinonasal	19 (3.7%)
CNS	15 (2.9%)
Skin**	14 (2.7%)
Salivary gland	12 (2.3%)
Eyelid/orbital	9 (1.7%)
Others	65 (12.5%)
Total	519 (100%)

* All missing cases were excluded.

** Excluding melanoma

**Table 3.** Leading sites of new cancer cases in AYAs by sex.

Male*		Female*	
Bone and soft tissue	2 8(20.0%)	Breast	203 (52.2%)
Nasopharynx	19 (13.6%)	Cervical	60 (15.4%)
Sinonasal	15 (10.7%)	Bone and soft tissue	39 (10.0%)
Colorectal	12 (8.6%)	Colorectal	14 (3.6%)
CNS	9 (6.4%)	Nasopharyngeal	10 (2.6%)
Skin**	9 (6.4%)	Salivary gland	8 (2.0%)
Salivary gland	4 (2.9%)	CNS	6 (1.5%)
Renal	4 (2.9%)	Thyroid	5 (1.3%)
Eyelid/orbital	4 (2.9%)	Eyelid/orbital	5 (1.3%)
Laryngeal	3 (2.1%)	Ovarian	5 (1.3%)
Others	33 (23.5%)	Others	34 (8.7%)
Total	140 (100.0%)	Total	389 (100.0%)

*All missing cases were excluded. **Excluding melanoma

**Table 4.** Leading sites of new cancer cases in AYAs by age group.

15–19*		20–29*		30–39*	
Bone and soft tissue	15 (46.9%)	Bone and soft tissue	23 (21.5%)	Breast	183 (48.2%)
CNS	3 (9.4%)	Breast	19 (17.8%)	Cervix	52 (13.7%)
Nasopharynx	3 (9.4%)	Colorectal	11 (10.3%)	Bone and soft tissue	29 (7.6%)
Colorectal	2 (6.3%)	Nasopharynx	11 (10.3%)	Sinonasal	16 (4.2%)
Lip and oral cavity	2 (6.3%)	Cervix	8 (7.5%)	Nasopharynx	15 (3.9%)
Thyroglossal duct cyst	1 (3.1%)	CNS	7 (6.5%)	Colorectal	13 (3.4%)
Salivary gland	1 (3.1%)	Skin**	4 (3.7%)	Skin**	10 (2.6%)
Breast	1 (3.1%)	Sinonasal	3 (2.8%)	Salivary gland	9 (2.4%)
Lymphoma	1 (3.1%)	Salivary gland	2 (1.9%)	Eyelid and orbital	8 (2.1%)
Nephroblastoma	1 (3.1%)	Lymphoma	2 (1.9%)	CNS	5 (1.3%)
Others	2 (6.3%)	Others	17 (15.9%)	Others	40 (10.5%)
Total	32 (100.0%)	Total	107 (100.0%)	Total	380 (100.0%)

*All missing cases were excluded. **Excluding melanoma

**Table 5.** Age and sex distribution of common cancers in AYAs.

Variables	Breast	Bone and soft tissue	Cervix	Nasopharynx	Colorectal	Sinonasal
Mean age (years)						
All	34.6 ± 3.8	27.5 ± 7.6	34.8 ± 3.8	29.2 ± 7.3	29.2 ± 6.3	32.7 ± 5.6
Males	--	26.5 ± 8.3	--	29.7 ± 8.0	30.9 ± 6.6	32.5 ± 6.3
Females	34.6 ± 3.8	28.2 ± 7.1	34.8 ± 3.8	28.2 ± 6.0	27.7 ± 5.9	33.5 ± 2.4
Age group						
15–19	1 (0.5%)	15 (22.4%)	0 (0.0%)	3 (10.3%)	2 (7.7%)	0 (0.0%)
20–29	19 (9.4%)	23 (34.3%)	8 (13.3%)	11 (37.9%)	11 (42.3%)	3 (15.8%)
30–39	183 (90.1%)	29 (43.3%)	52 (86.7%)	15 (51.7%)	13 (50.0%)	16 (84.2%)
Sex						
Males	0 (0.0%)	28 (41.8%)	0 (0.0%)	19 (65.5%)	12 (46.2%)	15 (78.9%)
Females	203 (100.0%)	39 (58.2%)	60 (100.0%)	10 (34.5%)	14 (53.8%)	4 (21.1%)
